# Problematic alcohol use among gay, bisexual, and other men who have sex with men in Canada: the role of proximal stressors and anxiety

**DOI:** 10.1186/s13011-024-00597-8

**Published:** 2024-02-28

**Authors:** Adhm Zahran, Sarah S. Dermody, Graham W. Berlin, Paolo A. Palma, Shayna Skakoon-Sparling, Syed W. Noor, Nathan J. Lachowsky, Daniel Grace, Joseph Cox, David M. Moore, Gilles Lambert, Terri H. Zhang, Milada Dvorakova, Jody Jollimore, Allan Lal, Trevor A. Hart

**Affiliations:** 1 Toronto Metropolitan University, Toronto, Canada; 2 University of Guelph, Guelph, Canada; 3 Louisiana State University Shreveport, Shreveport, LA USA; 4 University of Victoria, Victoria, BC Canada; 5 Community-Based Research Centre, Vancouver, BC Canada; 6 University of Toronto, Toronto, Canada; 7 McGill University, Montreal, QC Canada; 8 Direction Régionale de Santé Publique - Montréal, Montréal, QC Canada; 9 BC Centre for Excellence in HIV/AIDS, Vancouver, BC Canada; 10 University of British Columbia, Vancouver, BC Canada; 11 Institut National de Santé Publique du Québec, Montréal, QC Canada; 12 Canadian AIDS Treatment Information Exchange (CATIE), Toronto, Canada; 13 Research Institute of the McGill University Health Centre, Montreal, QC Canada

**Keywords:** Sexual minority stress, Anxiety, Depression, Alcohol use, Gay men

## Abstract

**Background:**

Gay, bisexual, and other men who have sex with men (GBM) report high rates of problematic alcohol use, anxiety, and depression. This may, in part, be due to stressors related to their sexual identity (i.e., minority stressors). However, few studies have examined both distal and proximal stressors, as well as the specific psychological mechanisms by which these stressors may be related to alcohol use outcomes, in a representative sample of GBM. We explored the relationship between distal and proximal stressors and alcohol use outcomes, as well as the role of anxiety and depression as potential mediators of these relationships.

**Methods:**

We analyzed the baseline data of 2,449 GBM from Engage, a cohort study of sexually active GBM recruited using respondent-driven sampling (RDS) in Montreal, Toronto, and Vancouver from February 2017 to August 2019. Using structural equation modeling, we examined the associations between distal minority stressors (i.e., experiences of heterosexist harassment, rejection, and discrimination), proximal minority stressors (i.e., internalized homonegativity, concerns about acceptance, concealment, and lack of affirmation), anxiety and depression, and alcohol consumption and alcohol use problems. RDS-adjusted analyses controlled for age, income, sexual orientation, ethnicity, recruitment city, and HIV serostatus.

**Results:**

There were positive direct associations between distal stress and proximal stress, anxiety, and depression, but not alcohol use outcomes. Proximal stress had a positive direct association with anxiety, depression, and alcohol use problems, but not alcohol consumption. Anxiety was positively associated with alcohol consumption and alcohol use problems. Depression was negatively associated with alcohol consumption but not alcohol use problems. Regarding indirect effects, distal stress was associated with alcohol use outcomes via proximal stress and anxiety, but not via depression.

**Conclusions:**

We found support for a minority stress model as it relates to alcohol use outcomes among GBM. Findings suggest that proximal minority stress and anxiety differentially impact the problematic alcohol use among GBM who experience heterosexist discrimination. Clinical providers should consider incorporating the treatment of proximal minority stressors and anxiety into existing alcohol interventions for GBM.

**Supplementary Information:**

The online version contains supplementary material available at 10.1186/s13011-024-00597-8.

## Background

Problematic alcohol use, including risky drinking (e.g., binge drinking, heavy drinking), having alcohol use-related problems (e.g., impacts on social or occupational functioning), or having a diagnosis of alcohol use disorder [1], is a significant public health burden. Problematic alcohol use incurs significant costs to healthcare and criminal justice systems in Canada [2, 3] and globally [4]. Among GBM, problematic alcohol use has been associated with increased risk of contracting human immunodeficiency virus (HIV) [5], increased neuroinflammation in GBM who are living with HIV [6], and higher rates of non-adherence to HIV pre-exposure prophylaxis (PrEP) and antiretroviral therapy (ART) medication [7, 8]. These risks are pertinent as GBM are disproportionately impacted by HIV [9]. Further, problematic alcohol use has been associated with other health and psychosocial problems among GBM, including adverse liver health and mood and anxiety disorders [10–12]. To date, few tailored GBM-specific interventions have demonstrated efficacy to reduce problematic alcohol use in this population [13]. Investigating how unique risk factors and related psychological mechanisms, such as sexual minority stressors, are associated with problematic alcohol use in GBM is needed to develop more effective interventions that prevent or treat problematic use and its harmful consequences.

Minority stress theory [14, 15] posits that GBM experience increased rates of negative health outcomes due to societal stigmatization of their sexual orientation. This stigmatization can manifest as external (i.e., distal) stressors, such as discrimination in workplace settings or interpersonal interactions [16]. These distal stressors can become proximal when GBM become distressed in anticipation of these stressors, leading to concerns about acceptance or through the internalization of negative societal evaluations (e.g., internalized homonegativity) [17]. These experiences of stigmatization have been reliably associated with poor mental health outcomes among GBM, including anxiety and depression [18, 19]. This may contribute to the disproportionately higher rates of psychiatric disorders, including alcohol use disorder, reported by GBM [20, 21].

Regarding anti-GBM stressors’ associations with problematic alcohol use, parts of the minority stress model have been tested with regards to alcohol use, and results have been somewhat mixed. Some studies have found that distal stressors like discrimination and structural stigma, and proximal stressors like internalized homonegativity, were positively associated with alcohol use outcomes [22–26]. Other studies have shown contradicting evidence; Lea et al. [27] found an inverse association between perceived stigma and alcohol use outcomes, and others [28, 29] found no significant direct associations between distal stressors and alcohol use outcomes. In addition, while some studies on minority stressors and alcohol use outcomes focus on older GBM [30, 31] most work examined younger groups of GBM (< 30 years old) and recruited convenience samples that were unable to correct for possible sampling biases, which limits validity and our ability to generalize these findings to the rest of the GBM population. There is a need to generalize findings to middle-aged and older GBM due to an ever-increasing aging population of GBM that are experiencing significant disparities in problematic alcohol use relative to their heterosexual peers [32, 33].

### Intervening psychological variables between minority stress and problematic alcohol use

Moreover, there is limited research on psychological distress (i.e., anxiety, depression) as an intervening variable in the relationship between sexual minority stressors and alcohol use outcomes. Moody et al. [34] found an indirect positive relationship via depression between internalized homonegativity and recent drug use in GBM, but did not examine distal stressors or alcohol use. Pesola et al. [35] found that sexual minority adolescents were more likely than heterosexual adolescents to report problematic alcohol use, and that depressed mood mediated the relationship between sexual orientation and alcohol use. This study, however, did not measure minority stressors and did not provide results specifically for GBM. Lastly, Livingston et al. [36] found an indirect and positive relationship via psychological distress (anxiety and depression) between distal minority stress and alcohol use outcomes. To our knowledge, no studies to date have examined intervening psychological distress variables in the associations of distal and proximal stressors and alcohol use outcomes in a large, community-representative sample of GBM. Further investigation into modifiable psychological mechanisms, such as anxiety or depression, could help identify targets for intervention for GBM with alcohol use- and comorbid mental health-related problems.

### The present study

Given the inconclusive evidence regarding the association between sexual minority stressors and alcohol use outcomes in GBM, the present study aims to apply a more comprehensive model that incorporates both distal and proximal minority stressors, as well as test how these variables may be associated with alcohol use outcomes indirectly through psychological distress. To accomplish this, we used structural equation modeling (SEM) to evaluate cross-sectional associations between measures of both proximal and distal minority stressors, anxiety and depression, and alcohol consumption and alcohol use problems. The structural equation models allowed for testing different indirect pathways to multiple alcohol outcomes simultaneously. By incorporating latent variables in these models, it was possible to represent constructs of interest with multiple related indicators, while reducing measurement error and maintaining model parsimony. Further, this study used a large, multi-city sample of adult GBM that adjusted for sampling biases, which provides greater generalizability compared to existing work.

We hypothesized that 1) distal minority stressors will be associated with proximal minority stressors, which will in turn be associated with alcohol use outcomes and 2) distal and proximal minority stressors will be indirectly associated with alcohol use outcomes through anxiety and depression. The aim of this study is to further increase the understanding of risk factors and mechanisms behind different forms of problematic alcohol use among GBM to help implement more targeted interventions.

## Methods

### Participants

A total of 2,449 GBM (aged 16 – 80 years, *M* = 36.79 years, *SD* = 12.84) were recruited as part of the Engage cohort study (Engage) from February 2017 to August 2019 from Toronto (*n* = 517), Montreal (*n* = 1,179), and Vancouver (*n* = 753) in Canada. Inclusion criteria for participants included being 16 + years of age, self-identifying as a man (inclusive of transgender men), able to read English or French (for Montreal participants only), living in one of the three study cities, and having engaged in sexual activity with another man in the past six months**.** See Table 1 for the demographic characteristics of participants by city.

**Table 1:** Crude and RDS adjusted demographics by city

	Montreal (*n* = 1,179)		Toronto (*n* = 517)		Vancouver (*n* = 753)	
Variable	Crude	RDS Adjusted	Crude	RDS Adjusted	Crude	RDS Adjusted
Age, in years, median (IQR)	34 (27–49)	33 (27–49)	31 (27–38)	29 (25–40)	32 (27–43)	31 (25–44)
	%	%	%	%	%	%
Sexual Orientation						
Gay	82.1	76.5	77.9	72.4	85.8	79.8
Bisexual	8.5	12.8	4.4	13.6	5.4	11.5
Queer	5.4	4.6	14.5	9.3	5.8	3.7
Pansexual	2.3	3.3	2.5	2.9	1.5	1.1
Two-Spirit	0.6	0.5	0.6	1.9	0.8	2.8
Others	1.1	2.3	0	0	0.7	1.1
Race/ethnicity						
White	76.9	70.6	64.9	59.9	66.0	54.6
Black	2.6	2.2	4.4	5.6	2.6	4.7
Latin American	8.2	10.1	7.7	8.4	7.7	10.4
East-Southeast Asian	1.9	1.9	8.3	10.4	14.7	17.9
Indigenous	0.8	1.2	0.6	2.2	2.9	3.9
South Asian	1.0	2.1	3.9	3.6	2.6	4.7
West Asian/ North African	4.4	7.1	3.1	3.6	0.8	0.4
Others	1.7	2.9	3.4	2.9	0.9	0.8
Mixed race/ethnicity	2.4	1.9	3.5	3.9	1.6	1.9
HIV Test Results						
Negative/unknown	81.8	85.8	80.6	77.8	82.4	79.6
Positive	18.2	14.2	19.4	22.2	17.5	20.4
Annual Income						
Less than $30,000	57.5	66.8	47.8	57.4	45.5	61.3
$30,000—$59,999	30.7	25.3	30.9	31.9	29.6	25.6
$60,000 or more	11.7	7.8	21.3	10.6	24.8	13.1

*RDS* respondent-driven sampling, *IQR* interquartile range, *N* = 2,449

Participants were recruited using respondent-driven sampling (RDS), a sampling method where initial respondents who were non-randomly selected (referred to as ‘seed’ participants) then recruited other eligible participants from their social networks. This form of chain referral sampling has been shown to decrease the bias present in convenience sampling and approximate probability sampling [37]. Details on the methodology of Engage, including the application of RDS, have been previously published [38–40]. The study was approved by research ethics boards at Toronto Metropolitan University, University of Toronto, St. Michael’s Hospital, University of Windsor, University of British Columbia, Providence Health Care, University of Victoria, Simon Fraser University, and the Research Institute-McGill University Health Centre. 

### Measures

#### Distal sexual minority stressors

The Heterosexist Harassment, Rejection, and Discrimination scale (HHRDS) [16] included 14 items that assessed participants’ frequency of experienced *harassment and rejection* (HR; e.g., “How many times have you been treated unfairly by your family because you are a gay/bisexual man?”), *workplace and school discrimination* (WD; e.g., “How many times have you been treated unfairly by teachers or professors because you are a gay/bisexual man?”), and *other discrimination* (OD; e.g., “How many times have you been treated unfairly by strangers because you are a gay/bisexual man?”). Items were scored on a 6-point Likert-type scale ranging from 1 (“Never”) to 6 (“Almost all of the time (> 70%)”), with higher scores indicating greater frequency of experienced harassment, rejection, and/or discrimination. In our sample, Cronbach’s alphas for the subscales ranged from 0.81 to 0.88. In structural equation models, the HHRDS-HR, -WD, and -OD were used as indicators for a latent variable of distal minority stress.

#### Proximal sexual minority stressors

The 12-item Lesbian, Gay, and Bisexual Identity scale (LGBIS) [17] assessed participants’ levels of *internalized homonegativity* (IH; e.g., “If it were possible, I would choose to be straight.”), *acceptance concerns* (AC; e.g., “I often wonder whether others judge me for my sexual orientation.”), *concealment motivation* (CM; e.g., “I prefer to keep my same-sex romantic relationships rather private.”), and *identity affirmation* (IDA; e.g., “I’m proud to be part of the LGB (Lesbian, Gay, Bisexual) community.”). Items are scored on a 6-point Likert-type scale ranging from 1 (“Disagree strongly”) to 6 (“Agree strongly”), with higher scores indicating higher levels of the given construct. In our sample, Cronbach’s alphas for the subscales ranged from 0.79 to 0.88. In structural equation models, the LGBIS-IH, -AC, -CM, and -IDA were used as indicators for a latent variable of proximal minority stress.

#### Alcohol use outcomes

##### Alcohol consumption

The Alcohol Use Disorders Identification Test-Consumption (AUDIT-C) [41] includes three items that screen for problematic use related to drinking frequency (e.g., “How often do you have a drink containing alcohol?”) and drink count (e.g., “How many standard drinks containing alcohol do you have on a typical day when you are drinking?”). This measure was designed and validated with a timeframe of the past 12 months [41]; however, no time frame was specified when it was administered in this study. Items are scored on a scale ranging from 0 to 4 points, with higher scores indicating greater consumption and likelihood for poor alcohol outcomes. In this sample, Cronbach’s alpha for the AUDIT-C was 0.76. Despite the AUDIT-C typically being used as a screening tool, scores in the current analyses were examined continuously (as opposed to applying cut-offs) as done previously by other studies [42, 43]. Our use of a continuous variable allowed us to examine alcohol use in a more nuanced way than a yes/no conceptualization would offer.

##### Alcohol use problems

The Alcohol Smoking and Substance Involvement Screening Test (ASSIST) [44, 45] includes eight items covering nine groups of substances (e.g., alcohol, tobacco, cannabis, stimulants, hallucinogens). Questions assess frequency as well as problematic use, including questions on cravings, control, and effects on social, occupational, and recreational functioning (e.g., “During the past six months how often has your use of the substances below led to health, social, legal or financial problems?”). Different sets of items are scored on different scales; total scores ranged from 0 to 39, with higher scores indicating more problematic alcohol use. A total alcohol score (ASSIST-Alcohol) was calculated summing only items for participants who reported alcohol use in the past six months or any lifetime use; all other participants were assigned a missing value. In this sample, Cronbach’s alpha was 0.83.

### Psychological distress

The Hospital Anxiety and Depression scale (HADS) [46] included seven items that assess anxiety (HADS-A subscale) and seven items that assess depression (HADS-D subscale). Items were scored on a 4-point Likert-type scale ranging from 0 to 3 points, with higher scores indicating more anxiety or depression symptoms. In our sample, Cronbach’s alphas for the HADS-A and HADS-D were 0.85 and 0.76, respectively. In structural equation models, each item on the HADS-A was used as an indicator for a latent variable of anxiety, and each item on the HADS-D was used as an indicator for a latent variable of depression.

### Statistical analyses

SEM analyses were conducted using lavaan (version 0.6–14) [47] in RStudio (version 2023.06.0 + 42). Age, income (multi-categorical; 12 categories ranging from $0 to $100,000 or more), ethnicity (GBM of colour versus white), sexual orientation (bisexual versus other sexual orientation), city where data were collected (dummy-coded; Toronto and Vancouver versus Montreal reference group), and HIV serostatus (HIV-negative/unknown versus HIV-positive) were included as covariates in all models. Mean scores were calculated only for participants who completed at least 80% of the items for each scale, and total scores were calculated only for participants with complete data. RDS weighting was applied for all analyses. See Table 2 for RDS-adjusted bivariate correlations among variables. Means, standard deviations, and bivariate correlations of all observed variables can be seen in the Supplementary Information (Additional File 1).

**Table 2:** Bivariate Correlations of Predictor and Outcome Variables

Variable	1	2	3	4	5
1. Distal Minority Stress^a^					
2. Proximal Minority Stress^a^	.156**				
3. Anxiety^a^	.345**	.151**			
4. Depression^a^	.307**	.288**	.664**		
5. AUDIT-C^b^	.032	-.042	.127**	-.031	
6. ASSIST-Alcohol^b^	.116*	.134**	.201**	.116**	.588**

*N* = 2,449. RDS adjusted values are shown

*AUDIT-C* Alcohol consumption, *ASSIST-Alcohol* alcohol use problems

*p<.05, **p<.01

^a^represents latent variables

^b^represents observed variables

Tests of assumptions included assessing linearity, multicollinearity, normality, and homoscedasticity for the variables of interest [48]. The comparative fit index (CFI), Tucker–Lewis Fit Index (TLI), root mean square error approximation (RMSEA), and standardized root-mean-square residual (SRMR) were used to evaluate overall SEM model fit. According to Hu and Bentler’s guidelines [49], model fit indices were targeted to have values of CFI ≥ 0.95, TLI ≥ 0.95, RMSEA ≤ 0.06, and SRMR ≤ 0.08. To avoid the possibility of rejecting a correctly specified model, χ^2^ was not used as an index of model fit due to the study’s large sample size and non-normality in the data, to which both the χ^2^ test is sensitive [48]. Full information maximum likelihood estimation with robust standard errors (Huber-White) [50, 51] as well as a Yuan-Bentler scaled test statistic [52] were used to accommodate partially missing data, non-normality, and heteroscedasticity [47, 53]^1^. Bootstrapped 95% confidence intervals were not used due to software limitations; the current version of lavaan was not capable of generating bootstrapped confidence intervals when applying sampling weights. Therefore, Sobel’s test [54] was used to calculate indirect associations.

## Results

### Measurement model

Model fit indices of the initial measurement model were CFI = 0.925, TLI = 0.915, RMSEA = 0.054 (95% CI [0.047, 0.061]), SRMR = 0.057. Modification indices (MIs) were examined one at a time to improve model fit, and only changes that were theoretically defensible [55] and that did not lead to evidence of model misfit were made. Three MIs were applied that involved adding residual covariances or cross loadings between subscales or items within a scale. More information on the MIs, as well as justification for applying said MIs, can be seen in the Supplementary Information (Additional File 1). After application of MIs, model fit improved to CFI = 0.963, TLI = 0.957, RMSEA = 0.038 (95% CI [0.031, 0.046]), SRMR = 0.050. This measurement model was maintained in the estimation of the structural model described below.

### Structural equation model

Model fit indices of the structural equation model were CFI = 0.934, TLI = 0.920, RMSEA = 0.040 (95% CI [0.035, 0.045]), SRMR = 0.049. Nine MIs were applied that involved adding residual covariances or cross loadings between subscales or items within a scale (see Supplementary Information; Additional File 1). Doing so improved model fit to CFI = 0.950, TLI = 0.937, RMSEA = 0.035 (95% CI [0.030, 0.041]), SRMR = 0.047.

### Direct associations

**Fig. 1:**
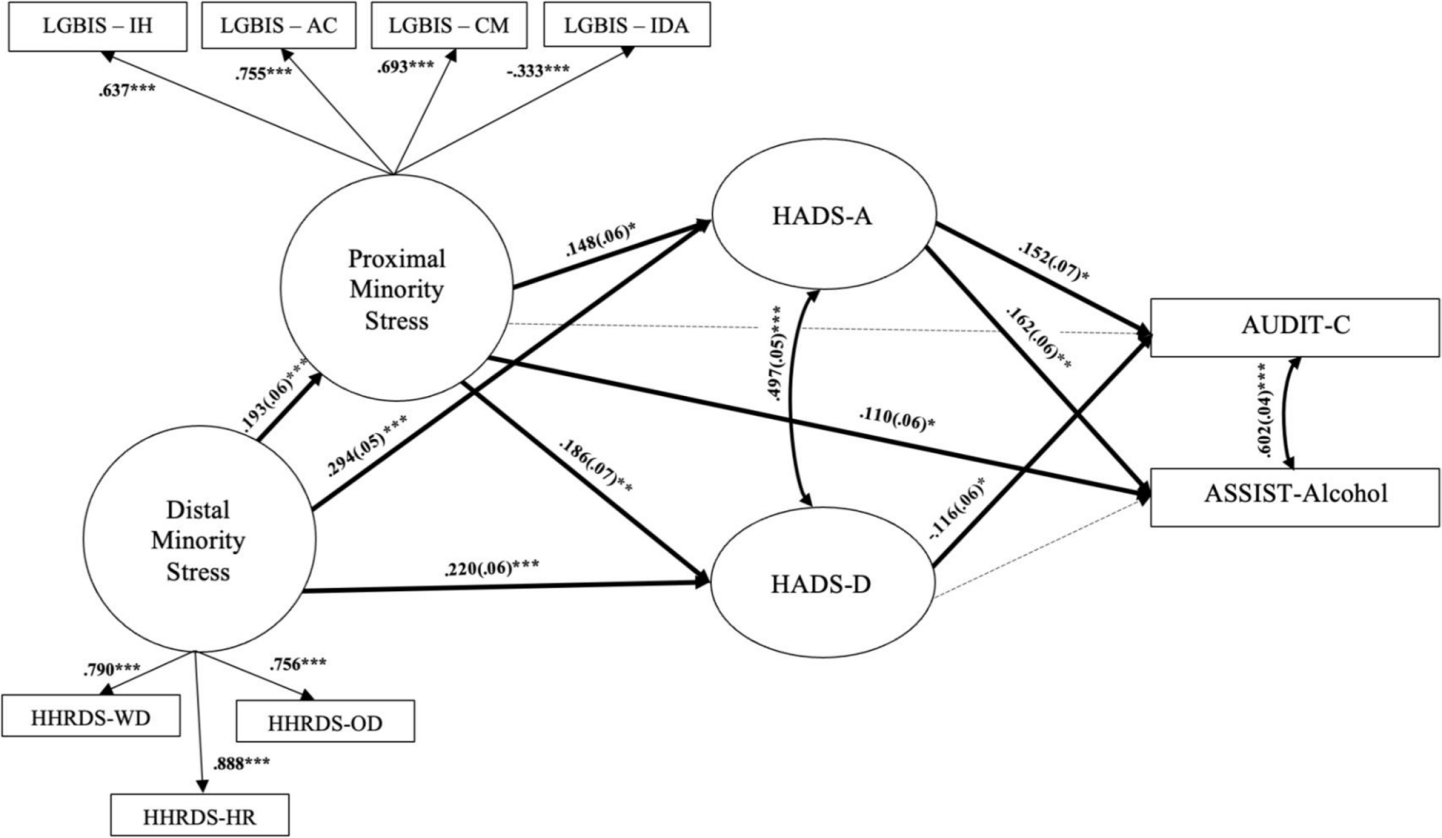
Estimated Structural Equation Model. Legend: Note. Standardized path coefficients and factor loadings (standard errors). *p < .05, **p < .01, ***p < .001. Dashed paths are non-significant. Factor loadings of the HADS-A and HADS-D indicator variables have been omitted from the diagram for clarity. Residuals, some residual covariances, and direct paths from distal minority stress to alcohol outcomes (two paths, both of which were statistically nonsignificant) have also been omitted for clarity. All indicators significantly loaded onto their corresponding latent variable. Standardized factor loadings for the depression latent variable ranged from .221 to .780, and for the anxiety latent variable ranged from .228 to .778. AUDIT-C *R* ^*2*^ = 12.3%, ASSIST-Alcohol *R* ^*2*^ = 7.7%. HHRDS-HR = harassment and rejection, HHRDS-WD = workplace and school discrimination, HHRDS-OD = other discrimination, LGBIS-IH = internalized homonegativity, LGBIS-AC = acceptance concerns, LGBIS-CM = concealment motivation, LGBIS-IDA = identity affirmation, HADS-A = anxiety, HADS-D = depression, AUDIT-C = alcohol consumption, ASSIST-Alcohol = alcohol use problems

Standardized path coefficients, standard errors, and factor loadings are shown in Fig. 1. There was a positive relationship between distal stressors, proximal stressors, and psychological distress such that GBM who experienced greater distal stress also experienced greater proximal stress, and GBM experiencing greater distal and proximal stress experienced greater anxiety and depression. Although distal minority stress was not significantly associated with either alcohol use outcomes, proximal minority stress was positively associated with ASSIST-Alcohol but was not significantly associated with AUDIT-C. Anxiety was positively associated with AUDIT-C and ASSIST-Alcohol. Depression was negatively associated with AUDIT-C, but was not significantly associated with ASSIST-Alcohol.

### Indirect associations

There were three significant indirect effects: 1) distal minority stress was positively associated with ASSIST-Alcohol via proximal minority stress, α*β* = 0.021, *SE* = 0.011, *p* = 0.048; 2) distal minority stress was positively associated with ASSIST-Alcohol via anxiety, α*β* = 0.048, *SE* = 0.019, *p* = 0.010; and 3) distal minority stress was positively associated with AUDIT-C via anxiety, α*β* = 0.045, *SE* = 0.021, *p* = 0.032. There was also a significant positive total indirect association from distal minority stress to ASSIST-Alcohol when summing the indirect associations via proximal minority stress, anxiety, and depression, α*β* = 0.081, *SE* = 0.024, *p* = 0.001. There were no significant indirect associations via depression, as well as from proximal minority stress to alcohol use outcomes via anxiety or depression.

### Post hoc examination of four outliers

Analyses revealed four outliers after examining their influence, leverage, and discrepancy using influence plots. However, to better understand the extent findings were impacted by these outliers, sensitivity analyses were conducted after removing the four outliers. Deleting the outliers reduced CFI below 0.95. Some previously significant effects in the original analyses were no longer significant; the direct association between proximal minority stress and ASSIST-Alcohol (*β* = 0.073, *p* = 0.087) as well as the related indirect association from distal minority stress to ASSIST-Alcohol via proximal stress (α*β* = 0.017, *p* = 0.100). However, three indirect associations that were marginally significant in the original analyses reached statistical significance: positive associations from proximal minority stress to both alcohol use outcomes via anxiety, which are consistent with study hypotheses; and a negative association from distal minority stress to alcohol consumption via depression, which is inconsistent with study hypotheses.

Therefore, although some results were marginal, the overall pattern of findings was similar with or without the outliers, with one exception. Further, all outliers were within the ranges of the corresponding variables (i.e., none had erroneous values). Thus, we operated under the assumption that they were natural variations in our community sample to avoid the possibility of removing true variability in the data, and we chose to retain the outliers in our main analyses. This decision on how to handle the outliers in our data is guided by the approach from Leys et al. [56], which emphasizes the importance of qualitative examination to determine whether to retain or exclude outliers from analyses. Previous studies using survey data have chosen to retain outliers using a similar approach [57–59].

## Discussion

In a large community sample of GBM across the three largest cities in Canada, we found support for the minority stress model as it relates to alcohol use outcomes. Heterosexist discrimination was associated with increased alcohol use problems separately via proximal minority stressors and anxiety. Heterosexist discrimination was also associated with increased alcohol consumption via anxiety. Although both heterosexist discrimination and proximal minority stressors were associated with increased depression, they were not associated with either alcohol consumption or alcohol use problems via depression.

The current findings are broadly consistent with previous data showing links between distal and proximal sexual minority stressors and psychological distress [18, 19]. The study results also extend previous work on sexual minority stress and alcohol use outcomes among GBM [22, 24, 25] by including measures of both distal and proximal minority stressors, multiple alcohol use outcomes (i.e., alcohol consumption and alcohol use problems), as well as recruiting a more representative sample of GBM. The current findings additionally add to the growing body of literature on the potential mechanisms involved in alcohol use outcomes among GBM [23, 26, 35, 36, 60] by examining indirect associations via depression and anxiety.

Of note, increased heterosexist discrimination was associated with increased problematic alcohol use due to either proximal stressors or anxiety, but not due to proximal stressors and then anxiety. These results suggest that proximal stressors and anxiety may differentially impact alcohol use outcomes of GBM who experience heterosexist discrimination. Further, there were varying findings depending on the alcohol use outcome; for example, proximal minority stressors were both directly and indirectly associated with increased alcohol use problems, but not alcohol consumption. Given these findings, in addition to previous research showing that measuring different alcohol outcomes could yield different results [61, 62], it is important to explore different measures of alcohol use to better recognize the nuances in findings.

We also found that depression was associated with decreased alcohol consumption, in addition to a lack of indirect effects from minority stressors to either alcohol use outcome via depression. These findings differ from some previous literature, which found that psychological distress was associated with greater alcohol use [35, 36]. This inconsistency may be attributed to differences in how psychological distress was operationalized across studies. For example, Livingston et al. [36] operationalized anxiety and depression as a single measure of psychological distress, instead of modeling them separately, as we did in the present study. Because Livingston et al. [36] used a combined measure, it is possible that only anxiety was positively associated with alcohol use outcomes in their study, but this effect was masked due to the combined variable they used. The association between anxiety and the alcohol use outcomes in our findings is logical due to alcohol’s effect of decreasing sympathetic nervous system activation [1]. This is an effect that GBM may purposefully seek to inhibit or cope with their feelings of anxiety [63] related to minority stressors. On the other hand, the negative association of depression and alcohol consumption could be due to depressed GBM being less involved in their community and, hence, less exposed to permissive substance use norms [64], resulting in them drinking less. Moody et al.’s study [34] lends support to this premise, which found that decreased gay community attachment was associated with decreased drug related problems. Alternatively, it is possible that only the more severely depressed GBM in our sample could be misusing alcohol as a method to cope with low mood [65]. However, this speculation is beyond the scope of the current work.

Given the different findings for anxiety versus depression in the present study, it is important that future work continues to investigate these psychological factors separately to explicate their differing associations with minority stressors and alcohol use outcomes. Including anxiety and depression together in one model as done in the present study helped partial out the overlapping effects of those highly correlated constructs and arrive at unique indirect effects.

### Implications

The current findings highlight the importance of considering the impact of proximal minority stressors and anxiety on alcohol use outcomes among GBM. Several psychological intervention trials have shown small, significant reductions in substance use among GBM; however, few have adapted them to target the minority stress experiences of GBM or have found large effect sizes for reducing problematic alcohol use (see Pantalone et al. [13] for a review). Given the associations found through proximal stressors and anxiety in the present study, specifically targeting minority stress-related cognitions in the context of GBM’s experiences of heterosexist discrimination may potentially boost future intervention efficacy when treating GBM for problematic alcohol use.

Some tailored interventions for GBM have been proposed that have shown some initially promising results (e.g., ESTEEM, Project PRIDE) [66–68], with participants reporting reductions in minority stressors, anxiety, and problematic alcohol use. However, several of these effects were nonsignificant possibly due to the studies being underpowered [66, 68], and one study [68] did not test the intervention against a control condition. Additionally, these were transdiagnostic interventions focusing on young GBM (16–35 years old) that targeted mental and behavioural outcomes broadly and not alcohol use specifically. Further investigation into how disorder-specific therapies could target proximal stressors and anxiety in GBM, and if that in turn reduces problematic alcohol use, is needed.

It is also important to acknowledge implications regarding public policy and clinical training programs. Policy makers and training programs should push to incorporate adapted evidence-based interventions in their substance use programs that consider the impact of minority stressors and anxiety when treating problematic alcohol use in GBM. Further, as all minority stress models originate from some form of distal, systemic stressor (e.g., heterosexism), it is essential for policy makers and institutions to increase efforts to reduce minority stressors in GBM from these structural sources, including the recent politicized opposition of sexual minorities and their rights [69]. Removing distal minority stressors for GBM at the structural level through policy, educational, and institutional reform is imperative to reduce their risk for negative health outcomes [70].

### Strengths, limitations, and future directions

Strengths of the present study include using a large sample of community-recruited GBM across the three most populous cities in Canada while adjusting for sampling biases using RDS-weights. These methods helped increase the generalizability and validity of estimates [71] to urban GBM across Canada. Further, the study tested distal and proximal minority stressors, multiple alcohol use outcomes, and multiple indirect pathways through anxiety and depression in a minority stress and alcohol use outcomes model for GBM. This allowed for a more comprehensive examination of minority stressors and their associations with specific mental health and alcohol use outcomes.

However, our cross-sectional design limits establishing causality and directionality. The hypothesized pathways were in line with the well supported self-medication hypothesis [72], which posits that people use substances to cope with psychological distress due to a lack of or an exhaustion of adaptive coping skills. However, previous research has also shown support for other directional pathways, including the substance-induced distress hypothesis [73] and the shared-vulnerability hypothesis [74]. For example, it is possible that GBM with pre-existing high anxiety or depression could be more vulnerable to minority stressors due to an exhaustion of coping mechanisms or due to interpretation biases [75]. The most likely temporal associations between anxiety, depression, and problematic alcohol use are still not fully understood by the present literature [76]. Therefore, it is imperative to examine future models longitudinally to better establish directionality and causality.

Future work may also benefit from including strength-based experiences, such as social support, community engagement, adaptive coping, or positive substance use motives, as GBM can have multiple and complex motives surrounding their substance use [77]. Additionally, future work may benefit from extending this model to include general psychological processes that mediate the link between stressors and mental health disorders in GBM, such as rumination, emotional regulation, or avoidant/positive coping [19, 78].

As the present study used continuous scores for alcohol consumption, future research could extend our model to examine stressors’ associations with the absence or presence of alcohol use disorders in GBM. Furthermore, alcohol outcomes were examined in isolation of other substance use in this study, which may not accurately capture the substance use patterns of many GBM [79, 80]. Future research may benefit from considering the impact of other substance use patterns when examining alcohol use outcomes among GBM. Additionally, as GBM are a heterogenous group with potentially unique risk factors based on their other minoritized identities (e.g., gay versus bisexual, Black versus Latino, cisgender versus gender-diverse) [81–83], future research should examine the intersectional experiences of GBM as it relates to alcohol use outcomes.

Finally, the present study included all outliers in the main analyses with the assumption that they were natural variations in the sample and to avoid the possibility of removing true variability in the data. However, running analyses without the outliers impacted some of our marginal findings, with one new finding that was inconsistent with study hypotheses. Despite our use of a large, multicity sample, there is a need to replicate our findings given some of the marginal associations between our variables. Nevertheless, the findings with and without the influential outliers correspond with consistent implications. That is, proximal stressors and anxiety are important in understanding the relationship between distal stressors and problematic alcohol use in GBM.

## Conclusions

The present study contributes to a growing body of research by examining the associations between sexual minority stressors and alcohol use outcomes in GBM, as well as the potential indirect pathways through psychological distress. The study found support for a minority stress model regarding GBM’s alcohol use outcomes as well some significant indirect associations via psychological processes, with divergent findings depending on the specific types of minority stressors, categories of psychological distress, and methods of measuring alcohol use outcomes. Our findings suggest that targeting proximal minority stress and anxiety may improve the treatment outcomes of alcohol use interventions for GBM. However, future work should test associations longitudinally to better determine causal pathways in the development of problematic alcohol use in GBM.

### Supplementary Information

**Supplementary Material 1.**

## Data Availability

The datasets generated and/or analyzed during the current study are not publicly available due to privacy concerns for this ongoing cohort study. Deidentified participant data used in this analysis are stored at the British Columbia Centre for Excellence in HIV/AIDS. For information regarding these databases, and related access, please contact the corresponding author (AZ).

## Abbreviations

GBM: Gay, bisexual, and other men who have sex with men
RDS: Respondent-driven sampling
HIV: Human immunodeficiency virus
SEM: Structural equation modeling
PrEP: Pre-exposure prophylaxis
ART: Antiretroviral therapy
HHRDS: Heterosexist harassment, rejection, and discrimination scale
HR: Harassment and rejection
WD: Workplace and school discrimination
OD: Other discrimination
LGBIS: Lesbian, gay, and bisexual identity scale
IH: Internalized homonegativity
AC: Acceptance concerns
CM: Concealment motivation
IDA: Identity affirmation
AUDIT-C: Alcohol use disorders identification test-Consumption
ASSIST: Alcohol smoking and substance involvement screening test
HADS-A: Hospital anxiety and depression scale-Anxiety
HADS-D: Hospital anxiety and depression scale-Depression
CFI: Comparative fit index
TLI: Tucker–Lewis fit index
RMSEA: Root mean square error approximation
SRMR: Standardized root-mean-square residual

## Footnotes

1 Given that the ASSIST-Alcohol was only shown to a subset of participants who used alcohol in their lifetime or in the past six months, the data were not missing at random. To address this, a sensitivity analysis included an additional binary covariate predicting the ASSIST-Alcohol outcome that consisted of a score of 0 (shown the ASSIST-Alcohol questions) versus 1 (not shown the ASSIST-Alcohol questions). Adding this covariate did not impact the associations among the variables; therefore, the full sample was retained with full information maximum likelihood estimation.
