# Supplementary material for: Problematic alcohol use among gay, bisexual, and other men who have sex with men in Canada: the role of proximal stressors and anxiety
Source: Subst Abuse Treat Prev Policy. 2024 Feb 28;19:16. doi: 10.1186/s13011-024-00597-8 (PMC10900570; doi:10.1186/s13011-024-00597-8)
Supplement: Supplementary file 1 — Supplementary Material 1. [file 13011_2024_597_MOESM1_ESM.docx]

**Supplementary Information for “Problematic Alcohol Use Among Gay, Bisexual, and Other Men Who Have Sex With Men in Canada: The Role of Proximal Stressors and Anxiety”**

**Modification Indices: Measurement Model**

After examination of modification indices (MIs), a residual covariance was added between internalized homonegativity and identity affirmation, which were two of the four indicator subscales of the proximal minority stress latent variable. This modification is theoretically consistent due to a methods effect possibly leading to residual covariance between the subscales of the same scale (Saris & Aalberts, 2003).

Another modification added was a cross-loading between the anxiety latent variable and an indicator scale item loading onto the depression latent variable (‘I feel as if I am slowed down’). This modification is theoretically consistent, as anxiety and depression were both measured using the same scale (i.e., Hospital Anxiety and Depression scale), anxiety and depression were highly correlated in our sample (*r* = .664), and anxiety and depression are highly comorbid in the population (Kessler et al., 2015).

The third and final modification added was a cross-loading between the depression latent variable and an indicator scale item loading onto the anxiety latent variable (‘I can sit at ease and feel relaxed’). Model fit indices: CFI = .963, TLI = .957, RMSEA = .038 (95% CI [.031, .046]), SRMR = .050.

**Modification Indices: Structural Equation Model**

After examination of MIs, a cross-loading was added between the anxiety latent variable and an indicator scale item loading onto the depression latent variable (‘I feel cheerful’). Other modifications added were residual covariances between scale items of the anxiety latent variable, between scale items of the depression latent variable, and between scale items across both the depression and anxiety latent variables. Three modifications were applied that added residual covariances between items of the anxiety latent variable, as well as three modifications that added residual covariances between items of the depression and anxiety latent variables.

Additional modifications included the residual covariance between workplace discrimination and other discrimination (two of the three indicator subscales of the distal minority stress latent variable), as well as the residual covariance between acceptance concerns and identity affirmation (two of the four indicator subscales of the proximal minority stress latent variable). Model fit indices: CFI = .950, TLI = .937, RMSEA = .035 (95% CI [.030, .041]), SRMR = .047.

**Structural Equation Model Without Outliers**

Model fit indices: CFI = .944, TLI = .930, RMSEA = .038 (95% CI [.033, .043]), SRMR = .048.


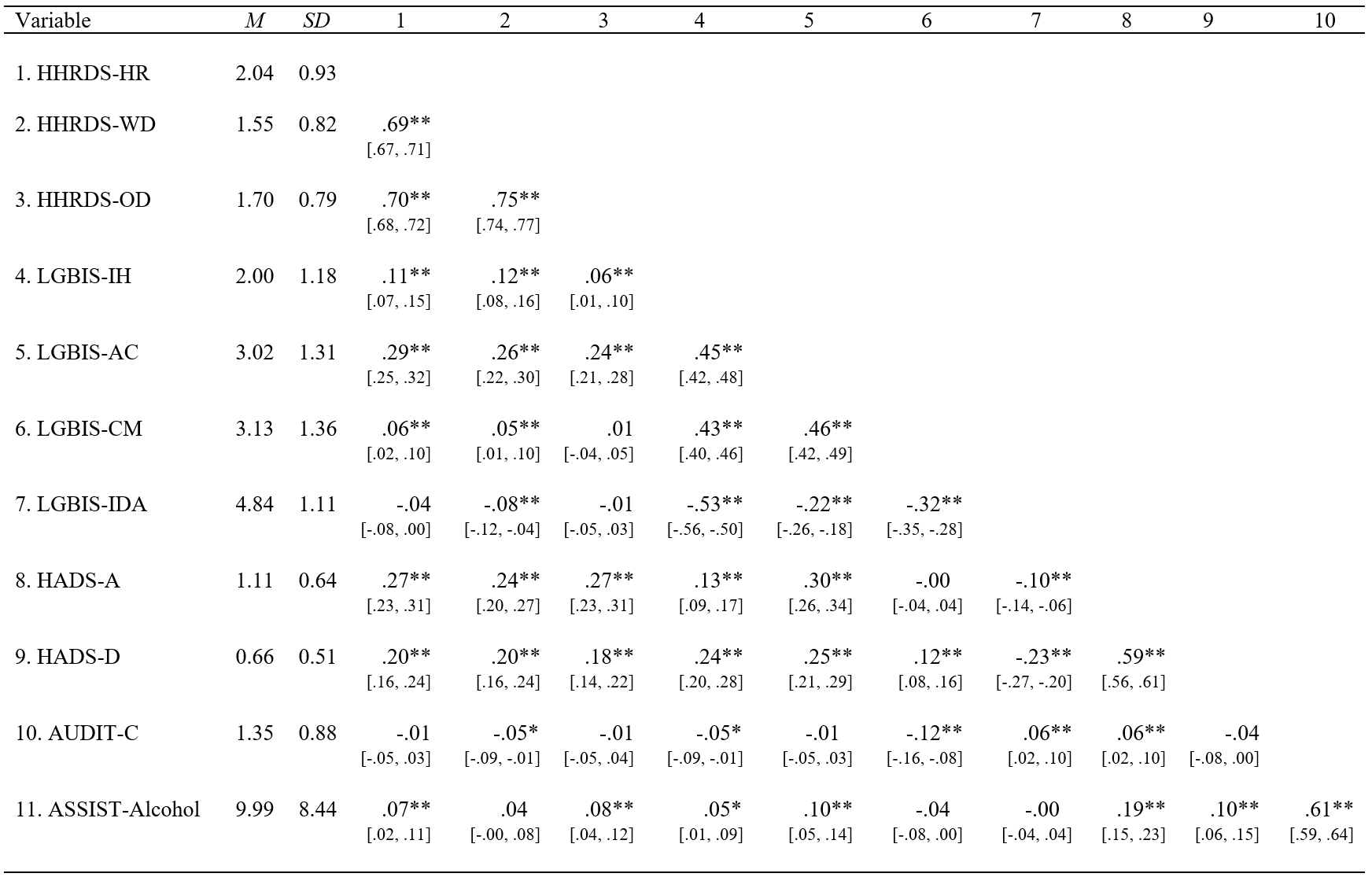


*Note.* Unadjusted values are shown. *M* and *SD* are used to represent mean and standard deviation, respectively. Values in square brackets indicate the 95% confidence interval for each correlation. **p* < .05, ***p* < .01. HHRDS-HR = harassment and rejection, HHRDS-WD = workplace and school discrimination, HHRDS-OD = other discrimination, LGBIS-IH = internalized homonegativity, LGBIS-AC = acceptance concerns, LGBIS-CM = concealment motivation, LGBIS-IDA = identity affirmation, HADS-A = anxiety, HADS-D = depression, AUDIT-C = alcohol consumption, ASSIST-Alcohol = alcohol use problems.

**References**

Kessler, R. C., Sampson, N. A., Berglund, P., Gruber, M. J., Al-Hamzawi, A., Andrade, L., Bunting, B., Demyttenaere, K., Florescu, S., de Girolamo, G., Gureje, O., He, Y., Hu, C., Huang, Y., Karam, E., Kovess-Masfety, V., Lee, S., Levinson, D., Medina Mora, M. E., … Wilcox, M. A. (2015). Anxious and non-anxious major depressive disorder in the World Health Organization World Mental Health Surveys. *Epidemiology and Psychiatric Sciences*, *24*(3), 210–226. <https://doi.org/10.1017/S2045796015000189>

Saris, W. E., & Aalberts, C. (2003). Different explanations for correlated disturbance terms in MTMM studies. *Structural Equation Modeling*, *10*(2), 193–213. [https://doi.org/10.1207/S15328007SEM1002_2](https://doi-org.ezproxy.lib.torontomu.ca/10.1207/S15328007SEM1002_2)
